# Specific Central Nervous System Medications Are Associated with Temporomandibular Joint Symptoms

**DOI:** 10.1155/2017/1026834

**Published:** 2017-07-16

**Authors:** John K. Drisdale III, Monica G. Thornhill, Alexandre R. Vieira

**Affiliations:** Department of Oral Biology, University of Pittsburgh School of Dental Medicine, Pittsburgh, PA, USA

## Abstract

**Aims:**

There is evidence of association between bruxism and the increasingly common central nervous system stimulants prescribed for attention deficit hyperactivity disorder (ADHD), as well as the selective serotonin reuptake inhibitors (SSRIs) often prescribed for depression or anxiety. However, the evidence is not clear on whether these medications inducing bruxism are directly associated with temporomandibular joint disorder (TMD). The aim of this work is to evaluate whether these medications are associated with TMD symptoms.

**Methods:**

Medical history and participant data were obtained for 469 patients from the University of Pittsburgh School of Dental Medicine, Dental Registry and DNA Repository, dating back to 2006. The chi-square test was used to determine any statistically significant associations.

**Results:**

There were no statistically significant associations between ADHD stimulant medications or SSRIs and reported TMD symptoms. However, there were significant differences seen between specific brands of medications and reported TMD symptoms. Individuals prescribed methylphenidate (Concerta) were less likely to report temporomandibular joint discomfort (*p* = 0.01). Conversely, individuals prescribed citalopram (Celexa) were more likely to report temporomandibular joint discomfort (*p* = 0.04).

**Conclusion:**

Signs and symptoms of temporomandibular joint dysfunction may be influenced by the use of certain medications prescribed for depression or attention deficit hyperactive disorder.

## 1. Introduction

As of 2012, more than 4.8 million Americans were prescribed stimulant medications to treat attention deficit hyperactivity disorder (ADHD) [1]. One out of ten Americans above the age of 12 are prescribed antidepressants, selective serotonin reuptake inhibitors (SSRIs) being the most common [2]. Central nervous system- (CNS-) stimulant ADHD medications have been associated with side effects such as bruxism [3] that can possibly cause TMD-like symptoms [4]. With the increasing use of stimulant ADHD medications, it is important to see if there is a relationship between these prescribed medications and TMD symptoms [5].

TMD can manifest in a variety of symptoms including pain or tenderness in the face muscles or jaw joint, a limited range of motion of the jaw, popping and clicking of the jaw, and headaches [6]. In addition to the adverse symptoms of TMD, treatment can often be extensive as well as expensive.

TMD symptoms are also more commonly seen in the female population. Of concern could be the recent increase in use of stimulant ADHD medications in women aged 26 to 34 [1]. The inherent predisposition to TMD symptoms in women combined with an increased use of ADHD medications could possibly put this patient population at an increased risk for developing TMD symptoms. Given this information, we hypothesize that there is an association between ADHD medications, as well as SSRIs, and the frequency of reported TMD symptoms.

## 2. Subjects and Methods

All subjects in this study were participants in the Dental Registry and DNA Repository of the University of Pittsburgh School of Dental Medicine [7–9]. All participants sought treatment at the University of Pittsburgh School of Dental Medicine dating back to September 2006. In April 2015, data from 4,959 individuals were queried from the registry and repository for this study and 469 individuals taking ADHD and SSRI medications were selected for this study. The records of these individuals were evaluated to determine if they had any TMD symptoms (pain or discomfort, cracking or clicking when opening the mouth).

Chi-square tests were used to determine associations between being prescribed any stimulant ADHD or SSRI medications and TMD symptoms. The same was performed for subjects prescribed any SSRI medication in search of a significant association between SSRIs and TMD symptoms. We used the likelihood ratio for comparisons when we violated the assumption of minimum expected cell count to be used for Pearson's chi-square. Alpha was established at 5%.

Standardized residuals were then compared to determine which medication in each category was responsible for a significantly different report rate of TMD symptoms.

## 3. Results

The mean age of the 469 participants was 46 years, with 38% of participants identifying as male (*N* = 179) and 62% female (*N* = 290). Furthermore, the majority of participants were White (*N* = 394; 84%). The remaining 16% were comprised of Black individuals (*N* = 52), Hispanics (*N* = 6), and other groups (*N* = 17).

There was a trend, albeit not statistically significant, for being prescribed ADHD medication and reporting TMD symptoms (*p* = 0.065). Likewise, there was no significant association between taking SSRIs and reporting TMD symptoms (*p* = 0.12). We found differences in occurrence of reported TMD symptoms between the different ADHD stimulant medications (*p* = 0.04, six degrees of freedom). The likelihood ratio of .010 suggested that there is at least one medication that is statistically significant among the others in regard to reported TMD symptoms. Further testing by means of finding the expected counts and adjusted residuals for each ADHD medication determined that Concerta was the statistically significant medication when compared to the others (Table 1). Additionally, taking the observed counts versus the expected counts in Table 1 into consideration also depicts that subjects prescribed Concerta are less likely to report TMD symptoms than subjects prescribed any other ADHD stimulant. There were no differences based on sex or ethnic background (data not shown).

Similarly, the group of SSRIs also showed that there is a medication with a significant difference in regard to reported TMD symptoms (*p* = 0.075, 10 degrees of freedom); thus the likelihood ratio is of .040. Celexa showed a difference in expected reporting of TMD symptoms when compared to the other SSRIs (Table 2). Additionally, taking the observed counts versus the expected counts in Table 2 into consideration also depicts that subjects prescribed Celexa are more likely to report TMD symptoms than subjects prescribed any other SSRI. There were no differences based on sex or ethnic background (data not shown).

Being prescribed both an ADHD medication and SSRI showed a trend, albeit not statistically significant, for reporting TMD symptoms (*p* = 0.074).

## 4. Discussion

The results show that there is no formal significant difference in TMD symptoms between those that are prescribed ADHD medications and those that are not prescribed ADHD medications. This same statement also holds true for subjects prescribed SSRIs and those not prescribed SSRIs. However, there does seem to be a significant difference* between* the different medications prescribed. Our data shows that subjects prescribed Concerta for ADHD are less likely to report TMD symptoms when compared to subjects taking other ADHD medications. It is also important to consider that our data shows that subjects taking the SSRI Celexa are more likely to report experiencing TMD symptoms than those prescribed an SSRI other than Celexa.

This could be clinically important when treating patients who are already at an increased risk for TMD. According to numerous studies, including the National Institute of Dental and Craniofacial Research, women are at higher risk than are men for developing TMD symptoms [6]. Since TMD is a multifactorial disorder [10], it may be wise to limit the number of contributing factors targeted for intervention. For example, a patient that is already predisposed to TMD may benefit from being prescribed Concerta versus other ADHD stimulants due to Concerta's lower frequency of reported TMD symptoms. Similarly, the same patient population that requires a SSRI prescription may benefit from a SSRI other than Celexa. Understanding better the contributing factors to this multifactorial disorder could prove beneficial to the patient's health and ultimately their quality of life.

In summary, we report here for the first time that specific ADNH and SSRI drugs may impact risks for developing TMD.

## Figures and Tables

**Table 1:** Comparison of the type of ADHD medication by the presence of TMD symptoms. The results show that Concerta is the ADHD medication that is significantly different compared to the rest.

	TMD diagnosis		Total
	No discomfort	Positive for discomfort	
*Type of ADHD medication*			
None			
Count	237	171	408
Expected count	243.6	164.4	408.0
% within type of medication	58.1%	41.9%	100.0%
Adjusted residual	−1.8	1.8	
Adderall			
Count	13	13	26
Expected count	15.5	10.5	26.0
% within type of medication	50.0%	50.0%	100.0%
Adjusted residual	−1.0	1.0	
Concerta			
Count	14	1	15
Expected count	9.0	6.0	15.0
% within type of medication	93.3%	6.7%	100.0%
Adjusted residual	**2.7**	−2.7	
Ritalin			
Count	11	4	15
Expected count	9.0	6.0	15.0
% within type of medication	73.3%	26.7%	100.0%
Adjusted residual	1.1	−1.1	
Vyvanse			
Count	3	0	3
Expected count	1.8	1.2	3.0
% within type of medication	100.0%	0.0%	100.0%
Adjusted residual	1.4	−1.4	
Adderall and Vyvanse			
Count	1	0	1
Expected count	.6	.4	1.0
% within type of medication	100.0%	0.0%	100.0%
Adjusted residual	.8	−.8	
Concerta and Ritalin			
Count	1	0	1
Expected count	.6	.4	1.0
% within type of medication	100.0%	0.0%	100.0%
Adjusted residual	.8	−.8	

Total			
Count	280	189	469
Expected count	280.0	189.0	469.0
% within type of medication	59.7%	40.3%	100.0%

Bold highlights the highest adjusted residual.

**Table 2:** Comparison of the type of SSRI medication by the presence of TMD symptoms. The results show that Celexa is the SSRI that is significantly different compared to the rest.

	TMD diagnosis		Total
	No discomfort	Positive for discomfort	
*Type of SSRI*			
None			
Count	80	42	122
Expected count	72.8	49.2	122.0
% within type of SSRI	65.6%	34.4%	100.0%
Adjusted residual	1.5	−1.5	
Celexa			
Count	30	35	65
Expected count	38.8	26.2	65.0
% within type of SSRI	46.2%	53.8%	100.0%
Adjusted residual	−2.4	**2.4**	
Lexapro			
Count	40	30	70
Expected count	41.8	28.2	70.0
% within type of SSRI	57.1%	42.9%	100.0%
Adjusted residual	−.5	.5	
Prozac			
Count	58	27	85
Expected count	50.7	34.3	85.0
% within type of SSRI	68.2%	31.8%	100.0%
Adjusted residual	1.8	−1.8	
Luvox			
Count	8	4	12
Expected count	7.2	4.8	12.0
% within type of SSRI	66.7%	33.3%	100.0%
Adjusted residual	.5	−.5	
Paxil			
Count	17	12	29
Expected count	17.3	11.7	29.0
% within type of SSRI	58.6%	41.4%	100.0%
Adjusted residual	−.1	.1	
Zoloft			
Count	41	34	75
Expected count	44.8	30.2	75.0
% within type of SSRI	54.7%	45.3%	100.0%
Adjusted residual	−1.0	1.0	
Lexapro and Zoloft			
Count	2	3	5
Expected count	3.0	2.0	5.0
% within type of SSRI	40.0%	60.0%	100.0%
Adjusted residual	−.9	.9	
Lexapro and Prozac			
Count	2	0	2
Expected count	1.2	.8	2.0
% within type of SSRI	100.0%	0.0%	100.0%
Adjusted residual	1.2	−1.2	
Lexapro and Paxil			
Count	0	2	2
Expected count	1.2	.8	2.0
% within type of SSRI	0.0%	100.0%	100.0%
Adjusted residual	−1.7	1.7	
Celexa and Zoloft			
Count	2	0	2
Expected count	1.2	.8	2.0
% within type of SSRI	100.0%	0.0%	100.0%
Adjusted residual	1.2	−1.2	

Total			
Count	280	189	469
Expected count	280.0	189.0	469.0
% within type of SSRI	59.7%	40.3%	100.0%

Bold highlights the highest adjusted residual.
